# Body weight variability and mortality in older adults: a nationwide population-based cohort study

**DOI:** 10.1038/s41598-026-55150-3

**Published:** 2026-05-28

**Authors:** Ji Yeon Seo, Yoo Min Han, Heesun Lee, Ju-Yeong Park, Ji Min Choi, Kyung-Do Han

**Affiliations:** 1 Department of Internal Medicine, Seoul National University College of Medicine, Seoul, Republic of Korea; 2 Department of Internal Medicine and Healthcare Research Institute, Healthcare System Gangnam Center, Seoul National University Hospital, Seoul, Republic of Korea; 3 Department of Statistics and Actuarial Science, Soongsil University, Seoul, Republic of Korea

**Keywords:** Body weight changes, Mortality, Aged, Cohort studies

## Abstract

Although body weight variability has been linked to adverse outcomes, its relationship with mortality in older adults remains unclear. We investigated the association between body weight variability and mortality in older adults using a nationwide cohort from the Korean National Health Insurance Service. Adults aged ≥ 75 years who underwent health checkups between 2012 and 2015 were followed until 2022. Body weight variability was assessed using the variability independent of the mean (VIM) based on at least four measurements. Participants were classified by VIM quartiles and weight-change categories. Mortality risks were estimated using multivariable Cox proportional hazards models. Among 616,696 participants, 195,585 deaths occurred during follow-up. Higher VIM was associated with increased all-cause mortality (adjusted HR for highest vs. lowest quartile, 1.62; 95% CI, 1.60–1.64) in a clear dose–response manner (*P* for trend < 0.001). This association was consistent across subgroups. High VIM was associated with increased mortality regardless of the direction of weight change. Higher VIM was independently associated with an increased risk of all-cause and cause-specific mortality among older adults. Body weight variability itself, rather than the direction of weight change, may serve as a relevant prognostic marker in this population. Further studies are needed to clarify the clinical implications of body weight variability in older adults.

**Supplementary Information:**

The online version contains supplementary material available at 10.1038/s41598-026-55150-3.

## Introduction

The global population aged 75 years and older has been increasing rapidly, leading to a substantial rise in healthcare expenditure^1,2^. Therefore, the extension of healthy life expectancy and prevention of chronic diseases have become important public health priorities^3^. Compared with younger adults, older adults exhibit distinct metabolic characteristics, including age-related changes in body composition, increased comorbidity burden, and reduced physiological resilience^4,5^.

Emerging evidence suggests that body weight variability is more closely associated with adverse health outcomes than single body mass index (BMI) measurements^6,7^. Weight variability, representing long-term dynamic changes in body weight, can result from intentional or unintentional causes^8,9^. Weight variability independent of the mean (VIM) has been proposed as a quantitative index of body weight fluctuation and has been associated with increased risk of cardiovascular disease, dementia, all-cause mortality^10–12^. However, most previous studies were conducted either in disease-specific cohorts or in general populations encompassing a wide range of ages, whereas in older adults, weight variability more often reflects unintentional changes due to underlying diseases, frailty, or medication use^13^.

With increasing prevalence of obesity and growing interest in weight management in older adults, obesity‑related metabolic and functional risks, particularly in the setting of age‑related muscle loss, underscore the importance of understanding the health implications of body weight fluctuation^14,15^. Despite the growing clinical relevance, large-scale studies analyzing the relationship between weight variability and mortality in older adult populations remain limited. Therefore, this study aimed to evaluate the association between long-term weight variability, assessed by VIM, and the risk of all-cause and cause-specific mortality in a large, nationwide cohort of individuals aged ≥ 75 years, and to assess whether weight variability itself has an independent effect on mortality risk regardless of the direction of weight change.

## Methods

### Study design and population

In this nationwide population-based cohort study, we analyzed data obtained from the Korean National Health Insurance Service (NHIS) health screening database. The NHIS provides mandatory health insurance to approximately 97% of the Korean population and offers standardized biennial health checkups to all insured individuals^16^.

We identified participants aged ≥ 75 years who underwent health checkups through Korean NHIS between 2012 and 2015 defined as the cohort enrollment window (n = 1,353,658). For each participant, the date of the first eligible health examination performed during this enrollment window was defined as the index date. The six years preceding the index date are referred to as the 6-year pre-index period. The following participants were excluded: (i) those who underwent fewer than three health examinations during the 6-year pre-index period (n = 705,992); (ii) those with any missing variables (n = 21,336); and (iii) those who died within the first year of follow-up (n = 9,634), to minimize the potential reverse causation from underlying disease. Follow-up was initiated on the index date and continued until December 31, 2022, or the date of death, whichever came first. The flow diagram of this study is shown in Supplementary Fig. S1 and the study timeline is illustrated in Supplementary Fig. S2.

This study was approved by the Ethics Committee at Seoul National University Hospital (Institutional Review Board No. E-2503-012-1618) and was conducted in accordance with the principles of the Declaration of Helsinki. The requirement for written informed consent was waived by the institutional review board because deidentified data from an existing clinical database was used in the analysis.

### Assessment of body weight variability

Participants’ height and body weight were measured at each health examination. At least 4 body weight measurements, including ≥ 3 prior measurements obtained during the 6-year pre-index period plus the index date measurement, were used in the analysis. Within-individual body weight variability was assessed using VIM. The VIM was calculated as 100 × SD/mean^β^, where SD and mean represent the standard deviation and average of body weight across repeated examinations, respectively, and β was derived from the regression coefficient of log(SD) on log(mean) for the entire study population. Eligible participants were stratified into quartiles based on VIM values. The corresponding VIM ranges were as follows: Q1, ≤ 1.29; Q2, 1.30–1.89; Q3, 1.90–2.76; and Q4, > 2.76 (Supplementary Table S1).

In additional analysis, participants were further classified according to the direction of body weight change to evaluate outcomes considering both variability and directional change. The percentage change in body weight was calculated as [(weight at the last examination − weight at the first examination)/weight at the first examination] × 100, and participants were subsequently classified into three groups based on the result: weight loss (< − 5%), stable weight (− 5% to + 5%), and weight gain (≥ + 5%). To examine the combined and independent effects of body weight variability and weight change on mortality, two models were applied. Model 1 used the stable weight group with VIM Q1–Q3 as the reference, while Model 2 evaluated the independent effect of VIM by comparing Q4 with Q1–Q3 within each weight change category.

### Definition of covariates

Demographic and clinical data, including age, sex, anthropometric measurements (BMI and waist circumference), blood pressure, lifestyle factors (smoking status, alcohol consumption, and physical activity), comorbidities (diabetes mellitus, hypertension, and dyslipidemia), and laboratory findings, were collected.

BMI was stratified into five categories in accordance with the Korean Society for the Study of Obesity (KSSO) clinical practice guidelines and the World Health Organization (WHO) Asia–Pacific recommendations: underweight (< 18.5 kg/m^2^), normal weight (18.5–22.9 kg/m^2^), overweight (23.0–24.9 kg/m^2^), obesity (25.0–29.9 kg/m^2^), and severe obesity (≥ 30.0 kg/m^2^), with BMI ≥ 25 kg/m^2^ defined as obesity^17,18^. Smoking status was categorized as non-, ex-, and current smoker. Alcohol consumption was categorized as non-drinker (no alcohol intake), mild drinker (> 0 and < 30 g/day for males; > 0 and < 20 g/day for females), and heavy drinker (≥ 30 g/day for males; ≥ 20 g/day for females)^19^. Regular physical activity was defined as moderate-intensity physical activity ≥ 5 days/week or vigorous exercise ≥ 3 days/week.

Hypertension was defined as International Classification of Diseases, 10th Revision (ICD-10) codes I10–I13 or I15 with antihypertensive medication, or as systolic/diastolic blood pressure ≥ 140/90 mmHg. Diabetes mellitus was defined as ICD-10 codes E11–E14 with antidiabetic treatment or a fasting glucose ≥ 126 mg/dL. Dyslipidemia was defined as ICD-10 code E78 with lipid-lowering treatment or a total cholesterol ≥ 240 mg/dL. Chronic kidney disease was defined as a history of end-stage renal disease (codes V001, V003, V005) or an estimated glomerular filtration rate calculated using the Chronic Kidney Disease Epidemiology Collaboration (CKD-EPI) equation < 60 mL/min/1.73 m^2^.

### Study outcomes

The primary outcome of this study was all-cause mortality. Secondary outcomes included cause-specific mortality and subgroup analyses stratified by the direction of body weight change. Information on mortality status and cause of death was obtained from the cause-of-death data provided by Statistics Korea. Causes of death were categorized according to ICD-10 codes, including deaths from infectious and parasitic diseases (A–B codes), neoplasms (C codes), endocrine and metabolic diseases (E codes), mental and behavioral disorders (F codes), nervous system diseases (G codes), circulatory system diseases (I codes), respiratory system diseases (J codes), digestive system diseases (K codes), genitourinary system diseases (N codes), and suicide (X60–X84 codes). Deaths from accidents or external causes were included in the all-cause mortality analysis but were not analyzed separately in the cause-specific mortality analyses.

### Statistical analysis

Characteristics at the index date were compared using chi-square tests or ANOVA, and multivariable Cox proportional hazards models were used to estimate hazard ratios (HRs) with 95% confidence intervals (CI) for outcomes across quartiles of body weight variability, defined as VIM, using the lowest quartile (Q1) as the reference. The proportional hazards assumption was assessed graphically using log-minus-log survival plots for major covariates. Follow-up time since the index date was used as the time scale.

Subgroup analyses were performed according to age, sex, lifestyle factors, anthropometric measures, comorbidities, and body weight change categories (loss, stable, gain), with interaction tests assessing heterogeneity across groups. Kaplan–Meier survival curves were generated to visualize the mortality differences across VIM, and log-rank tests were used to assess statistical significance between the groups. *P*-values of < 0.05 were considered statistically significant. Statistical analyses were performed using SAS version 9.4 (SAS Institute Inc., Cary, NC, USA).

## Results

### Demographic and clinical characteristics

Among 1,353,658 individuals aged ≥ 75 years who underwent health checkups through the Korean NHIS between 2012 and 2015, 616,696 were included in the analysis after excluding those with fewer than three prior checkups, missing covariate data, or death within the first year of follow-up (Supplementary Fig. S1). The clinical characteristics of the total cohort and each VIM quartile group are summarized in Table 1. The mean age of participants was 78.0 ± 2.9 years, and the median follow-up duration was 7.5 years (IQR, 6.2–8.7 years).

**Table 1:** Characteristics at the index date according to the body weight variability.

	Total (n = 616,696)	VIM of body weight			
		Q1 (n = 154,608)	Q2 (n = 153,854)	Q3 (n = 154,058)	Q4 (n = 154,176)
Age (yr)	78.0 ± 2.9	77.8 ± 2.7	77.9 ± 2.8	78.1 ± 2.9	78.4 ± 3.2
Sex (Male)	297,775 (48.3)	76,440 (49.4)	72,765 (47.3)	75,396 (48.9)	73,174 (47.5)
Low income^a^	92,044 (14.9)	22,345 (14.5)	23,346 (15.2)	22,968 (14.9)	23,385 (15.2)
Height (cm)	156.2 ± 9.2	156.6 ± 9.0	155.9 ± 9.2	156.3 ± 9.2	156.0 ± 9.5
Weight (kg)	57.6 ± 9.8	58.9 ± 9.1	57.4 ± 9.6	57.6 ± 9.8	56.4 ± 10.6
BMI (kg/m^2^)	23.5 ± 3.1	24.0 ± 2.9	23.6 ± 3.0	23.5 ± 3.1	23.1 ± 3.5
WC (cm)	83.0 ± 8.6	83.7 ± 8.1	82.7 ± 8.4	82.9 ± 8.6	82.7 ± 9.2
Obesity^b^	192,516 (31.2)	55,138 (35.7)	47,399 (30.8)	47,139 (30.6)	42,840 (27.8)
Smoking status					
Non-smoker	458,895 (74.4)	114,595 (74.1)	115,452 (75.0)	113,681 (73.8)	115,167 (74.7)
Ex-smoker	112,297 (18.2)	29,726 (19.2)	27,428 (17.8)	28,352 (18.4)	26,791 (17.4)
Current smoker	45,504 (7.4)	10,287 (6.7)	10,974 (7.1)	12,025 (7.8)	12,218 (7.9)
Drinking status					
Non	494,008 (80.1)	120,945 (78.2)	122,542 (79.7)	123,107 (79.9)	127,414 (82.6)
Mild	103,307 (16.8)	28,924 (18.7)	26,472 (17.2)	25,755 (16.7)	22,156 (14.4)
Heavy	19,381 (3.1)	4,739 (3.1)	4,840 (3.2)	5,196 (3.4)	4,606 (3.0)
Regular exercise	104,328 (16.9)	31,156 (20.2)	27,187 (17.7)	25,265 (16.4)	20,720 (13.4)
Diabetes	139,649 (22.6)	31,685 (20.5)	32,145 (20.9)	35,476 (23.0)	40,343 (26.2)
Dyslipidemia	238,032 (38.6)	61,798 (40.0)	59,637 (38.8)	59,129 (38.4)	57,468 (37.3)
Hypertension	435,896 (70.7)	108,578 (70.2)	106,826 (69.4)	108,695 (70.6)	111,797 (72.5)
Chronic kidney disease	156,186 (25.3)	36,661 (23.7)	36,653 (23.8)	39,033 (25.3)	43,839 (28.4)
Fasting glucose (mg/dL)	104.0 ± 24.7	103.6 ± 23.1	103.4 ± 23.5	104.0 ± 24.7	104.8 ± 27.3
Systolic BP (mmHg)	130.3 ± 15.5	130.7 ± 15.1	130.5 ± 15.4	130.3 ± 15.5	129.9 ± 15.9
Diastolic BP (mmHg)	76.9 ± 9.8	76.7 ± 9.6	76.9 ± 9.7	76.9 ± 9.9	76.9 ± 10.1
Total cholesterol (mg/dL)	188.5 ± 38.5	189.7 ± 37.7	189.7 ± 38.2	188.6 ± 38.7	186.0 ± 39.2
HDL-C (mg/dL)	52.5 ± 14.1	52.1 ± 14.2	52.7 ± 13.9	52.6 ± 14.1	52.6 ± 14.0
LDL-C (mg/dL)	110.2 ± 34.8	111.4 ± 34.3	111.2 ± 34.7	110.2 ± 35.0	108.0 ± 35.1
Triglycerides (mg/dL)	114.3 (114.2–114.4)	116.3 (116.1–116.6)	114.7 (114.5–115.0)	114.2 (113.9–114.4)	112.0 (111.8–112.3)

Continuous variables are presented as mean ± standard deviation or geometric mean (95% confidence interval), and categorical variables are presented as n (%).

BMI, body mass index; BP, blood pressure; HDL-C, high-density lipoprotein cholesterol; LDL-C, low-density lipoprotein cholesterol; VIM, variability independent of the mean; WC, waist circumference.

^a^Low income is defined as the lowest income quartile or receiving medical aid.

^b^Obesity is defined as BMI ≥ 25 kg/m^2^.

In comparison with participants in Q1, those in Q4 tended to be older (78.4 vs. 77.8 years), included a greater proportion of women (52.5% vs. 50.6%), and showed higher prevalences of diabetes (26.2% vs. 20.5%), hypertension (72.5% vs. 70.2%), and chronic kidney disease (28.4% vs. 23.7%). Conversely, participants in Q4 showed a lower mean BMI (23.1 vs. 24.0 kg/m^2^), a lower prevalence of obesity (27.8% vs. 35.7%), and a less frequent engagement in regular physical activity (13.4% vs. 20.2%). Overall, higher weight variability was accompanied by a greater comorbidity burden despite lower BMI.

### All-cause and cause-specific mortality according to body weight variability

There were 195,585 cases of all-cause mortality during the median follow-up period of 7.5 years in the entire cohort. Table 2 demonstrates the risk of all-cause and cause-specific mortality of participants according to VIM. After adjusting for potential confounders (Model 3), all-cause mortality in Q4 was 62% higher compared with Q1 (adjusted HR [aHR], 1.62; 95% CI, 1.60–1.64). Mortality risk increased progressively with higher VIM quartiles across all adjusted models.

**Table 2:** Risk of all-cause and specific-cause of death in relation to body weight variability.

Cause of death	WT VIM	N	Event	IR^a^	Model 1^b^	Model 2^c^	Model 3^d^
All-cause	Q1	154,608	38,416	34	1.00 (Reference)	1.00 (Reference)	1.00 (Reference)
	Q2	153,854	43,564	39.3	1.16 (1.14–1.17)	1.14 (1.13–1.16)	1.12 (1.10–1.13)
	Q3	154,058	49,800	45.8	1.35 (1.34–1.37)	1.30 (1.28–1.32)	1.26 (1.24–1.28)
	Q4	154,176	63,805	62	1.86 (1.84–1.88)	1.73 (1.70–1.75)	1.62 (1.60–1.64)
	*p* value				< 0.001	< 0.001	< 0.001
Infectious and parasitic diseases	Q1	154,608	1,157	1	1.00 (Reference)	1.00 (Reference)	1.00 (Reference)
	Q2	153,854	1,412	1.3	1.25 (1.15–1.35)	1.23 (1.13–1.32)	1.18 (1.09–1.27)
	Q3	154,058	1,700	1.6	1.53 (1.42–1.65)	1.47 (1.37–1.59)	1.40 (1.30–1.51)
	Q4	154,176	2,287	2.2	2.22 (2.07–2.38)	2.04 (1.90–2.19)	1.85 (1.72–1.99)
	*p* value				< 0.001	< 0.001	< 0.001
Neoplasms	Q1	154,608	11,870	10.5	1.00 (Reference)	1.00 (Reference)	1.00 (Reference)
	Q2	153,854	12,191	11	1.05 (1.02–1.07)	1.05 (1.03–1.08)	1.04 (1.02–1.07)
	Q3	154,058	13,214	12.1	1.16 (1.13–1.19)	1.13 (1.11–1.16)	1.12 (1.10–1.15)
	Q4	154,176	14,189	13.8	1.32 (1.29–1.35)	1.29 (1.25–1.32)	1.26 (1.23–1.29)
	*p* value				< 0.001	< 0.001	< 0.001
Endocrine and metabolic diseases	Q1	154,608	974	0.9	1.00 (Reference)	1.00 (Reference)	1.00 (Reference)
	Q2	153,854	1,186	1.1	1.24 (1.14–1.35)	1.22 (1.13–1.33)	1.19 (1.10–1.30)
	Q3	154,058	1,467	1.4	1.57 (1.45–1.70)	1.51 (1.40–1.64)	1.38 (1.27–1.50)
	Q4	154,176	2,090	2	2.40 (2.22–2.59)	2.23 (2.06–2.41)	1.80 (1.67–1.94)
	*p* value				< 0.001	< 0.001	< 0.001
Mental and behavioral disorders	Q1	154,608	354	0.3	1.00 (Reference)	1.00 (Reference)	1.00 (Reference)
	Q2	153,854	465	0.4	1.34 (1.17–1.54)	1.30 (1.13–1.49)	1.22 (1.07–1.41)
	Q3	154,058	482	0.4	1.42 (1.24–1.63)	1.33 (1.15–1.52)	1.25 (1.09–1.43)
	Q4	154,176	853	0.8	2.70 (2.38–3.06)	2.33 (2.06–2.64)	2.09 (1.85–2.37)
	*p* value				< 0.001	< 0.001	< 0.001
Nervous system diseases	Q1	154,608	1,593	1.4	1.00 (Reference)	1.00 (Reference)	1.00 (Reference)
	Q2	153,854	1,828	1.6	1.17 (1.10–1.25)	1.15 (1.08–1.23)	1.11 (1.04–1.19)
	Q3	154,058	2,201	2	1.45 (1.36–1.54)	1.39 (1.30–1.48)	1.33 (1.25–1.42)
	Q4	154,176	3,212	3.1	2.28 (2.15–2.43)	2.09 (1.97–2.22)	1.95 (1.84–2.08)
	*p* value				< 0.001	< 0.001	< 0.001
Circulatory system diseases	Q1	154,608	7,746	6.9	1.00 (Reference)	1.00 (Reference)	1.00 (Reference)
	Q2	153,854	9,159	8.3	1.21 (1.17–1.24)	1.18 (1.15–1.22)	1.16 (1.12–1.19)
	Q3	154,058	10,526	9.7	1.42 (1.38–1.46)	1.35 (1.31–1.39)	1.31 (1.27–1.35)
	Q4	154,176	14,095	13.7	2.04 (1.98–2.10)	1.85 (1.80–1.90)	1.73 (1.68–1.78)
	*p* value				< 0.001	< 0.001	< 0.001
Respiratory system diseases	Q1	154,608	5,478	4.9	1.00 (Reference)	1.00 (Reference)	1.00 (Reference)
	Q2	153,854	6,555	5.9	1.22 (1.18–1.27)	1.21 (1.17–1.26)	1.15 (1.11–1.19)
	Q3	154,058	7,772	7.1	1.48 (1.43–1.54)	1.42 (1.37–1.47)	1.34 (1.29–1.39)
	Q4	154,176	10,825	10.5	2.22 (2.15–2.30)	2.04 (1.97–2.11)	1.84 (1.79–1.91)
	*p* value				< 0.001	< 0.001	< 0.001
Digestive system diseases	Q1	154,608	1,024	0.9	1.00 (Reference)	1.00 (Reference)	1.00 (Reference)
	Q2	153,854	1,225	1.1	1.22 (1.12–1.33)	1.20 (1.10–1.30)	1.18 (1.08–1.28)
	Q3	154,058	1,435	1.3	1.46 (1.35–1.58)	1.40 (1.30–1.52)	1.36 (1.26–1.48)
	Q4	154,176	1,826	1.8	1.99 (1.85–2.15)	1.84 (1.71–1.99)	1.74 (1.61–1.87)
	*p* value				< 0.001	< 0.001	< 0.001
Genitourinary system diseases	Q1	154,608	1,000	0.9	1.00 (Reference)	1.00 (Reference)	1.00 (Reference)
	Q2	153,854	1,140	1	1.16 (1.07–1.27)	1.14 (1.05–1.24)	1.14 (1.04–1.24)
	Q3	154,058	1,409	1.3	1.47 (1.36–1.60)	1.41 (1.30–1.53)	1.36 (1.26–1.48)
	Q4	154,176	1,993	1.9	2.25 (2.09–2.43)	2.05 (1.90–2.21)	1.87 (1.73–2.02)
	*p* value				< 0.001	< 0.001	< 0.001
Suicide	Q1	154,608	706	0.6	1.00 (Reference)	1.00 (Reference)	1.00 (Reference)
	Q2	153,854	686	0.6	0.99 (0.89–1.10)	1.01 (0.91–1.12)	0.99 (0.89–1.10)
	Q3	154,058	798	0.7	1.17 (1.06–1.30)	1.17 (1.06–1.30)	1.15 (1.04–1.28)
	Q4	154,176	816	0.8	1.27 (1.15–1.40)	1.29 (1.16–1.42)	1.24 (1.12–1.38)
	*p* value				< 0.001	< 0.001	< 0.001

IR, incidence rate; WT VIM, weight variability independent of the mean.

^a^Incidence rate per 1,000 person-years.

^b^Model 1 was unadjusted.

^c^Model 2 was adjusted for age, sex, income, smoking status, alcohol consumption, and regular exercise.

^d^Model 3 was adjusted for age, sex, income, smoking status, alcohol consumption, regular exercise, diabetes mellitus, dyslipidemia, hypertension, chronic kidney disease, and index weight.

**Fig. 1:**
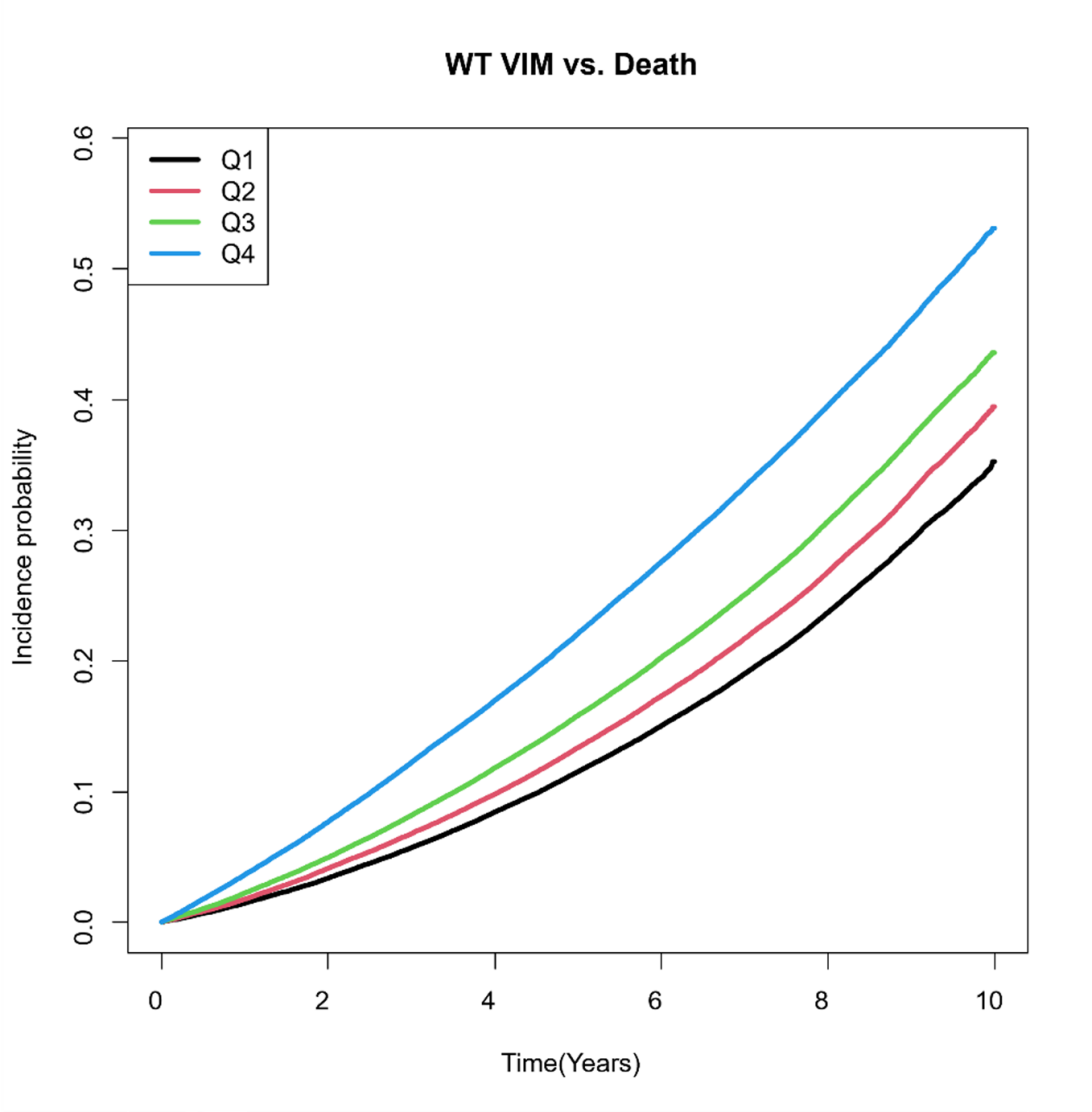
Kaplan–Meier curve of overall mortality rate by the body weight variability. Log Rank *p*-value < 0.0001.

The association between weight variability and mortality was most pronounced for deaths from mental and behavioral disorders, nervous system diseases, genitourinary system diseases, infectious and parasitic diseases, and respiratory system diseases, whereas the associations were weaker for neoplasms and suicide. Kaplan–Meier survival curves demonstrated a progressively increased risk of all-cause death from Q1 to Q4 (log-rank *P* < 0.0001; Fig. 1).

### Subgroup analysis of mortality risk according to body weight variability

Subgroup analyses were performed to evaluate whether the association between body weight variability and mortality differed across clinical characteristics (Table 3). VIM was consistently associated with elevated mortality across all subgroups. The association was stronger in participants who were < 85 years, women, non-smokers, non-drinkers, had dyslipidemia, or no diabetes. When stratified by BMI, the association between VIM and mortality was most pronounced in the normal BMI group (BMI 18.5–24.9, aHR 1.61), whereas relatively weaker associations were observed in the underweight group (BMI < 18.5, aHR 1.48) and the severe obesity group (BMI ≥ 30, aHR 1.46; *P* for interaction = 0.003).

**Table 3:** Subgroup analysis of all-cause mortality risk according to quartiles of body weight variability.

Subgroup	VIM group	N	Event	IR^a^	Model 3^b^	*p* for interaction
Age						0.013
Age < 85	Q1	150,891	36,222	32.8	1 (Ref)	
	Q2	149,335	40,715	37.6	1.12 (1.10–1.13)	
	Q3	148,755	46,245	43.7	1.26 (1.24–1.28)	
	Q4	146,462	58,006	58.5	1.63 (1.60–1.65)	
Age ≥ 85	Q1	3,717	2,194	97.3	1 (Ref)	
	Q2	4,519	2,849	106.5	1.10 (1.04–1.16)	
	Q3	5,303	3,555	118.5	1.21 (1.15–1.28)	
	Q4	7,714	5,799	150.3	1.51 (1.44–1.58)	
Sex						< 0.001
Male	Q1	76,440	24,825	46.2	1 (Ref)	
	Q2	72,765	26,758	53.5	1.11 (1.09–1.13)	
	Q3	75,396	31,009	61.4	1.25 (1.23–1.27)	
	Q4	73,174	37,093	81.2	1.56 (1.53–1.58)	
Female	Q1	78,168	13,591	23.0	1 (Ref)	
	Q2	81,089	16,806	27.6	1.14 (1.11–1.16)	
	Q3	78,662	18,791	32.2	1.28 (1.26–1.31)	
	Q4	81,002	26,712	46.6	1.72 (1.68–1.75)	
BMI						0.003
< 18.5	Q1	4,094	1,725	62.9	1 (Ref)	
	Q2	5,914	2,666	68.7	1.05 (0.99–1.12)	
	Q3	6,644	3,316	79.4	1.17 (1.10–1.24)	
	Q4	11,591	6,815	101.5	1.48 (1.40–1.56)	
18.5–22.9	Q1	51,347	14,662	39.7	1 (Ref)	
	Q2	60,184	18,909	44.0	1.10 (1.08–1.13)	
	Q3	61,541	22,032	51.4	1.25 (1.22–1.27)	
	Q4	66,036	29,727	68.8	1.61 (1.58–1.64)	
23–24.9	Q1	44,029	10,351	32.0	1 (Ref)	
	Q2	40,357	10,609	36.2	1.12 (1.09–1.15)	
	Q3	38,734	11,733	42.3	1.28 (1.24–1.31)	
	Q4	33,709	12,810	55.5	1.61 (1.57–1.65)	
25–29.9	Q1	51,583	10,922	28.6	1 (Ref)	
	Q2	43,915	10,596	33.0	1.14 (1.11–1.17)	
	Q3	43,116	11,753	37.7	1.27 (1.24–1.30)	
	Q4	37,724	12,855	48.8	1.58 (1.54–1.62)	
≥ 30	Q1	3,555	756	29.0	1 (Ref)	
	Q2	3,484	784	30.9	1.06 (0.96–1.17)	
	Q3	4,023	966	33.3	1.10 (1.00–1.21)	
	Q4	5,116	1,598	44.8	1.46 (1.34–1.59)	
Smoking status						< 0.001
Non, ex-smoker	Q1	144,321	34,041	32.1	1 (Ref)	
	Q2	142,880	38,493	37.1	1.12 (1.11–1.14)	
	Q3	142,033	43,858	43.4	1.27 (1.25–1.29)	
	Q4	141,958	56,813	59.5	1.65 (1.63–1.67)	
Current smoker	Q1	10,287	4,375	63.5	1 (Ref)	
	Q2	10,974	5,071	70.0	1.06 (1.02–1.11)	
	Q3	12,025	5,942	76.9	1.17 (1.13–1.22)	
	Q4	12,218	6,992	94.7	1.39 (1.34–1.45)	
Alcohol consumption						< 0.001
Non-drinker	Q1	120,945	28,841	32.5	1 (Ref)	
	Q2	122,542	33,486	37.7	1.12 (1.10–1.14)	
	Q3	123,107	38,715	44.3	1.27 (1.25–1.29)	
	Q4	127,414	51,978	60.9	1.64 (1.62–1.67)	
Mild–heavy drinker	Q1	33,663	9,575	39.6	1 (Ref)	
	Q2	31,312	10,078	45.6	1.11 (1.08–1.14)	
	Q3	30,951	11,085	51.6	1.23 (1.20–1.26)	
	Q4	26,762	11,827	67.0	1.52 (1.48–1.57)	
Regular exercise						0.070
No	Q1	123,452	31,684	35.2	1 (Ref)	
	Q2	126,667	36,772	40.3	1.11 (1.10–1.13)	
	Q3	128,793	42,668	47.0	1.26 (1.24–1.28)	
	Q4	133,456	56,430	63.6	1.62 (1.60–1.64)	
Yes	Q1	31,156	6,732	29.4	1 (Ref)	
	Q2	27,187	6,792	34.5	1.14 (1.10–1.18)	
	Q3	25,265	7,132	39.5	1.26 (1.21–1.30)	
	Q4	20,720	7,375	51.8	1.58 (1.53–1.63)	
Diabetes						< 0.001
No	Q1	122,923	28,706	31.7	1 (Ref)	
	Q2	121,709	32,492	36.7	1.11 (1.09–1.13)	
	Q3	118,582	36,414	43.0	1.26 (1.24–1.28)	
	Q4	113,833	45,156	58.6	1.64 (1.61–1.66)	
Yes	Q1	31,685	9,710	43.5	1 (Ref)	
	Q2	32,145	11,072	49.7	1.14 (1.11–1.17)	
	Q3	35,476	13,386	55.5	1.25 (1.22–1.28)	
	Q4	40,343	18,649	72.1	1.56 (1.52–1.60)	
Hypertension						0.324
No	Q1	46,030	10,754	31.9	1 (Ref)	
	Q2	47,028	12,511	36.7	1.10 (1.08–1.13)	
	Q3	45,363	13,985	43.4	1.25 (1.22–1.28)	
	Q4	42,379	16,813	59.0	1.63 (1.59–1.67)	
Yes	Q1	108,578	27,662	35.0	1 (Ref)	
	Q2	106,826	31,053	40.4	1.12 (1.10–1.14)	
	Q3	108,695	35,815	46.8	1.26 (1.24–1.28)	
	Q4	111,797	46,992	63.1	1.61 (1.59–1.64)	
Dyslipidemia						0.025
No	Q1	92,810	24,732	36.5	1 (Ref)	
	Q2	94,217	28,389	41.9	1.11 (1.09–1.13)	
	Q3	94,929	32,407	48.4	1.24 (1.22–1.26)	
	Q4	96,708	41,904	65.3	1.60 (1.57–1.62)	
Yes	Q1	61,798	13,684	30.3	1 (Ref)	
	Q2	59,637	15,175	35.2	1.13 (1.11–1.16)	
	Q3	59,129	17,393	41.5	1.29 (1.26–1.32)	
	Q4	57,468	21,901	56.5	1.66 (1.62–1.69)	
Chronic kidney disease						0.165
No	Q1	117,947	26,521	30.5	1 (Ref)	
	Q2	117,201	30,291	35.5	1.12 (1.10–1.14)	
	Q3	115,025	33,664	40.8	1.25 (1.23–1.27)	
	Q4	110,337	41,603	55.1	1.62 (1.59–1.64)	
Yes	Q1	36,661	11,895	45.9	1 (Ref)	
	Q2	36,653	13,273	52.2	1.11 (1.08–1.13)	
	Q3	39,033	16,136	61.4	1.28 (1.25–1.30)	
	Q4	43,839	22,202	80.8	1.62 (1.58–1.65)	

BMI, body mass index; CI, confidence interval; IR, incidence rate; Ref, reference; VIM, variability independent of the mean.

All subgroup characteristics were based on values at the index date.

^a^Incidence rate per 1,000 person-years.

^b^Hazard ratios (95% confidence intervals) adjusted for age, sex, income, smoking status, alcohol consumption, regular exercise, diabetes mellitus, dyslipidemia, hypertension, chronic kidney disease, and index weight.

### Mortality risk according to weight variability and weight change

Table 4 presents the combined effects of weight variability and weight change on mortality risk. In Model 1, all weight-change groups showed higher all-cause mortality compared with the reference (stable weight with VIM Q1–Q3). The highest risk was observed in Q4 of the weight-loss group (aHR 1.52, 95% CI 1.50–1.54), followed by Q4 of the weight-gain group (≥ 5%; HR 1.42, 95% CI 1.39–1.45), and Q4 of the weight-stable group (–5% to 5%; HR 1.41, 95% CI 1.38–1.44). In Model 2, elevated mortality risk in VIM Q4 was consistently observed across all weight-change categories, demonstrating that high weight variability was associated with increased mortality independent of the direction of body weight change.

**Table 4:** Subgroup analysis of mortality risk by quartiles of body weight variability and categories of weight change.

Outcome	Weight change	VIM group	N	Event	IR^a^	Model 1^b^	Model 2^c^
All-cause	< − 5%	Q1–Q3	105,962	34,048	45.3	1.12 (1.10–1.13)	1.00 (Reference)
		Q4	99,321	43,094	65.9	1.52 (1.50–1.54)	1.36 (1.34–1.38)
	–5 to 5%	Q1–Q3	313,471	85,706	37.9	1.00 (Reference)	1.00 (Reference)
		Q4	27,137	10,513	56.8	1.41 (1.38–1.44)	1.41 (1.38–1.44)
	≥ 5%	Q1–Q3	43,087	12,026	38.8	1.07 (1.05–1.09)	1.00 (Reference)
		Q4	27,718	10,198	53.6	1.42 (1.39–1.45)	1.33 (1.30–1.37)
	*p* for interaction					< 0.001	0.003
Infectious and parasitic diseases	< − 5%	Q1–Q3	105,962	1,207	1.6	1.18 (1.10–1.27)	1.00 (Reference)
		Q4	99,321	1,603	2.5	1.69 (1.58–1.80)	1.42 (1.32–1.53)
	–5 to 5%	Q1–Q3	313,471	2,665	1.2	1.00 (Reference)	1.00 (Reference)
		Q4	27,137	330	1.8	1.44 (1.29–1.62)	1.44 (1.29–1.62)
	≥ 5%	Q1–Q3	43,087	397	1.3	1.13 (1.02–1.26)	1.00 (Reference)
		Q4	27,718	354	1.9	1.65 (1.48–1.85)	1.46 (1.26–1.68)
	*p* for interaction					< 0.001	0.954
Neoplasms	< − 5%	Q1–Q3	105,962	8,444	11.2	1.04 (1.02–1.07)	1.00 (Reference)
		Q4	99,321	9,002	13.8	1.23 (1.20–1.26)	1.18 (1.14–1.21)
	–5 to 5%	Q1–Q3	313,471	25,311	11.2	1.00 (Reference)	1.00 (Reference)
		Q4	27,137	2,607	14.1	1.19 (1.14–1.24)	1.19 (1.14–1.24)
	≥ 5%	Q1–Q3	43,087	3,520	11.4	1.05 (1.02–1.09)	1.00 (Reference)
		Q4	27,718	2,580	13.6	1.18 (1.13–1.23)	1.12 (1.06–1.18)
	*p* for interaction					< 0.001	0.136
Endocrine and metabolic diseases	< − 5%	Q1–Q3	105,962	960	1.3	1.08 (1.00–1.16)	1.00 (Reference)
		Q4	99,321	1,408	2.2	1.51 (1.41–1.61)	1.40 (1.29–1.52)
	–5 to 5%	Q1–Q3	313,471	2,383	1.1	1.00 (Reference)	1.00 (Reference)
		Q4	27,137	326	1.8	1.43 (1.27–1.60)	1.43 (1.27–1.60)
	≥ 5%	Q1–Q3	43,087	284	0.9	0.99 (0.87–1.12)	1.00 (Reference)
		Q4	27,718	356	1.9	1.74 (1.55–1.94)	1.76 (1.51–2.06)
	*p* for interaction					< 0.001	0.036
Mental and behavioral disorders	< − 5%	Q1–Q3	105,962	352	0.5	1.02 (0.90–1.16)	1.00 (Reference)
		Q4	99,321	617	0.9	1.89 (1.69–2.11)	1.85 (1.62–2.10)
	–5 to 5%	Q1–Q3	313,471	826	0.4	1.00 (Reference)	1.00 (Reference)
		Q4	27,137	114	0.6	1.57 (1.29–1.90)	1.57 (1.29–1.90)
	≥ 5%	Q1–Q3	43,087	123	0.4	1.13 (0.93–1.36)	1.00 (Reference)
		Q4	27,718	122	0.6	1.86 (1.53–2.25)	1.65 (1.28–2.12)
	*p* for interaction					< 0.001	0.355
Nervous system diseases	< − 5%	Q1–Q3	105,962	1,600	2.1	1.20 (1.13–1.27)	1.00 (Reference)
		Q4	99,321	2,281	3.5	1.89 (1.79–1.99)	1.58 (1.48–1.68)
	–5 to 5%	Q1–Q3	313,471	3,550	1.6	1.00 (Reference)	1.00 (Reference)
		Q4	27,137	492	2.7	1.64 (1.49–1.80)	1.64 (1.49–1.80)
	≥ 5%	Q1–Q3	43,087	472	1.5	1.00 (0.91–1.10)	1.00 (Reference)
		Q4	27,718	439	2.3	1.53 (1.39–1.69)	1.53 (1.35–1.75)
	*p* for interaction					< 0.001	0.687
Circulatory system diseases	< − 5%	Q1–Q3	105,962	7,285	9.7	1.15 (1.11–1.18)	1.00 (Reference)
		Q4	99,321	9,653	14.8	1.61 (1.57–1.65)	1.41 (1.37–1.45)
	–5 to 5%	Q1–Q3	313,471	17,576	7.8	1.00 (Reference)	1.00 (Reference)
		Q4	27,137	2,269	12.3	1.46 (1.40–1.53)	1.46 (1.40–1.53)
	≥ 5%	Q1–Q3	43,087	2,570	8.3	1.10 (1.05–1.14)	1.00 (Reference)
		Q4	27,718	2,173	11.4	1.45 (1.39–1.52)	1.33 (1.25–1.40)
	*p* for interaction					< 0.001	0.025
Respiratory system diseases	< − 5%	Q1–Q3	105,962	5,373	7.2	1.11 (1.08–1.15)	1.00 (Reference)
		Q4	99,321	7,448	11.4	1.65 (1.60–1.69)	1.48 (1.43–1.53)
	–5 to 5%	Q1–Q3	313,471	12,712	5.6	1.00 (Reference)	1.00 (Reference)
		Q4	27,137	1,726	9.3	1.57 (1.50–1.66)	1.57 (1.50–1.66)
	≥ 5%	Q1–Q3	43,087	1,720	5.6	1.05 (1.00–1.10)	1.00 (Reference)
		Q4	27,718	1,651	8.7	1.64 (1.56–1.73)	1.56 (1.46–1.67)
	*p* for interaction					< 0.001	0.080
Digestive system diseases	< − 5%	Q1–Q3	105,962	970	1.3	1.15 (1.06–1.24)	1.00 (Reference)
		Q4	99,321	1,243	1.9	1.59 (1.48–1.70)	1.38 (1.27–1.50)
	–5 to 5%	Q1–Q3	313,471	2,381	1.1	1.00 (Reference)	1.00 (Reference)
		Q4	27,137	302	1.6	1.45 (1.29–1.63)	1.45 (1.29–1.63)
	≥ 5%	Q1–Q3	43,087	333	1.1	1.05 (0.94–1.18)	1.00 (Reference)
		Q4	27,718	281	1.5	1.39 (1.22–1.57)	1.32 (1.13–1.55)
	*p* for interaction					< 0.001	0.644
Genitourinary system diseases	< − 5%	Q1–Q3	105,962	1,002	1.3	1.32 (1.23–1.43)	1.00 (Reference)
		Q4	99,321	1,377	2.1	1.86 (1.74–1.99)	1.41 (1.30–1.53)
	–5 to 5%	Q1–Q3	313,471	2,249	1	1.00 (Reference)	1.00 (Reference)
		Q4	27,137	317	1.7	1.53 (1.36–1.72)	1.53 (1.36–1.72)
	≥ 5%	Q1–Q3	43,087	298	1	1.00 (0.89–1.13)	1.00 (Reference)
		Q4	27,718	299	1.6	1.46 (1.29–1.65)	1.46 (1.24–1.71)
	*p* for interaction					< 0.001	0.509
Suicide	< − 5%	Q1–Q3	105,962	504	0.7	1.06 (0.96–1.18)	1.00 (Reference)
		Q4	99,321	501	0.8	1.17 (1.05–1.30)	1.10 (0.97–1.25)
	–5 to 5%	Q1–Q3	313,471	1,476	0.7	1.00 (Reference)	1.00 (Reference)
		Q4	27,137	157	0.9	1.28 (1.08–1.51)	1.28 (1.08–1.51)
	≥ 5%	Q1–Q3	43,087	210	0.7	1.09 (0.95–1.26)	1.00 (Reference)
		Q4	27,718	158	0.8	1.30 (1.10–1.53)	1.18 (0.96–1.46)
	*P* for interaction					0.001	0.356

IR, incidence rate; VIM, variability independent of the mean.

Weight change was defined as the difference between the first and last weight measurements during four consecutive health examinations.

Both model 1 and 2 were adjusted for age, sex, income, smoking status, alcohol consumption, regular exercise, diabetes mellitus, dyslipidemia, hypertension, chronic kidney disease, and index weight.

^a^Incidence rate per 1,000 person-years.

^b^Model 1: Overall comparison (reference: stable weight, Q1–Q3 VIM).

^c^Model 2: Within-group comparison (reference: Q1–Q3 VIM within each weight change category).

In cause-specific analyses, deaths from endocrine and metabolic diseases showed the highest risk in VIM Q4 among the weight-gain group, while deaths from circulatory system diseases showed the highest risk in VIM Q4 among the stable weight group (*P* for interaction < 0.05 in Model 2). No significant interactions were observed for other causes.

## Discussion

In this large nationwide cohort of older adults, all-cause mortality was significantly higher in individuals with greater weight variability, and mortality risk increased stepwise from the lowest to the highest VIM quartile for both all-cause and cause-specific deaths. Higher VIM was consistently associated with elevated mortality across clinical subgroups, with stronger associations among participants aged < 85 years, women, non-smokers, non-drinkers, and those with dyslipidemia. This association was significant in every BMI category, being most pronounced in the normal BMI group. Additionally, higher VIM was associated with increased mortality across all weight change categories, regardless of the direction. To our knowledge, this is the first large-scale study to comprehensively evaluate the relationship between body weight variability and both all-cause and cause-specific mortality in older adults.

Several studies have investigated the association between body weight change and mortality in older adults. A meta-analysis published in 2021 showed that weight loss (HR 1.59, 95% CI: 1.45–1.74), weight gain (HR 1.10, 95% CI: 1.02–1.17), and weight fluctuation (HR 1.66, 95% CI: 1.28–2.15) were all associated with an increased risk of all-cause mortality^20^. Similarly, a study in older adults from the United States and Australia found that weight loss was associated with higher all-cause and cause-specific mortality; in that study, men with more than a 10% decrease in body weight had a 3.89-fold higher risk of all-cause mortality (95% CI, 2.93–5.18) and women had a 2.14-fold higher risk (95% CI, 1.58–2.91) compared with those with stable weight^21^.

However, these studies were primarily conducted in Western populations, included some that relied on self-reported weight data, and assessed the net change in body weight or BMI (i.e., the difference between the final and baseline measurements) rather than dynamic changes over time. In contrast, our study used VIM, which captures the dynamic fluctuation of body weight over time, providing a more comprehensive assessment of long-term weight variability than a simple net change in body weight. The present study extends previous evidence by confirming, in a large nationwide population-based cohort of older adults in East Asia, that dynamic body weight fluctuations, independent of mean body weight, are associated with an increased risk of both all-cause and cause-specific mortality.

Although the underlying mechanisms remain incompletely elucidated, repeated weight fluctuation may promote chronic inflammation, metabolic dysregulation, and impaired adaptive homeostasis through adipose tissue remodeling and oxidative stress. These processes may increase vulnerability to cardiovascular disease, frailty, cognitive decline, and ultimately mortality in older adults^22–26^. However, in older adults, body weight variability may also reflect underlying subclinical illness, frailty, declining functional status, or other age-related health deterioration rather than directly causing mortality. Therefore, the observed associations should be interpreted with caution.

In our study, participants with higher body weight variability had lower BMI but a higher prevalence of chronic diseases. Although cardiometabolic diseases are more common among individuals with obesity, this paradoxical finding may be explained by unintentional weight changes driven by underlying illness, frailty, or deteriorating physical status rather than intentional weight control in the highest VIM quartile group^27^.

A clear dose–response relationship was observed, with mortality risk increasing stepwise from the lowest to the highest VIM quartile across all causes of death. Although hazard ratios were slightly attenuated after adjustment for multiple confounding variables, the associations remained statistically significant, supporting the role of body weight variability as an independent risk factor for mortality in older adults. Notably, the association was most pronounced for deaths from mental and behavioral disorders, such as depression, dementia, and alcohol use disorders. Previous studies have also reported associations between body weight instability and dementia risk, as well as depressive symptoms and impaired physical function, suggesting a potential link between body weight instability and adverse mental health outcomes^12,28^.

Interestingly, in subgroup analyses, the impact of high weight variability on mortality was more pronounced among participants aged < 85 years, women, those with normal-range BMI, non-smokers, non-drinkers, and those without diabetes—groups that are generally considered healthier. This finding suggests that body weight variability may serve as an early marker of physiological instability, making these individuals more susceptible to mortality despite the absence of conventional risk factors.

In contrast, the attenuated association observed in participants with diabetes or with extreme BMI may be explained by several potential mechanisms. One possible explanation is that diabetes or extreme BMI, both well-established risk factors for increased mortality, may mask the additional effect of body weight variability^29,30^. Moreover, diabetes itself may act as a potential driver of body weight fluctuations through poor glycemic control and glucose-lowering drug use, complicating the assessment of an independent association between VIM and mortality in individuals with diabetes^31^.

The effect of VIM on mortality did not differ according to the presence of hypertension, chronic kidney disease, or regular exercise status. The lack of modification by hypertension or chronic kidney disease may be explained by their limited influence on body weight fluctuation compared with diabetes. For regular exercise, although the relative risk associated with VIM was comparable between exercisers and non-exercisers, absolute incidence rates were consistently lower in regular exercisers across all VIM quartiles, suggesting that weight variability and physical activity may both contribute independently to survival in older adults.

Overall, these findings suggest that weight stability may be particularly important for low-risk older adults, while optimal management of comorbid diseases, such as diabetes, remains essential for minimizing mortality risk. In addition, regular physical activity may provide further survival benefit across all levels of weight variability.

Notably, weight gain was also associated with increased mortality in our study. Although weight gain has been considered relatively less harmful than weight loss in older adults—a phenomenon referred to as the “obesity paradox”—such weight increases do not necessarily indicate healthy gains in skeletal muscle mass. Instead, this weight gain may reflect underlying pathologic conditions, including fluid retention secondary to heart failure or renal dysfunction, decreased physical activity with functional decline, or increased adiposity accompanied by age-related muscle loss (sarcopenic obesity)^13–15^. Furthermore, reverse causation cannot be entirely excluded, as subclinical illness may affect both changes in body weight and mortality risk, despite the exclusion of deaths occurring within the first year of follow-up.

From a clinical perspective, our study highlights the potential relevance of body weight variability in older adults. Body weight variability remained independently associated with increased mortality regardless of BMI category or direction of weight change. Although modest weight reduction is generally recommended for individuals with cardiometabolic diseases, our results suggest that excessive weight fluctuation—intentional or unintentional—may be associated with higher mortality risk in older adults. These findings highlight the need for further investigation into the clinical implications of body weight variability in older adults.

This study has several limitations. First, the study population comprised exclusively Korean older adults, which may limit the generalizability of our findings to other ethnicities or age groups^32^. Asians generally have a higher proportion of body fat than Western populations at a similar BMI^33^. Such ethnic differences in body composition and fat distribution may lead to differences in study outcomes across populations. Second, owing to the study’s observational design, a causal relationship between body weight variability and mortality cannot be established. In older adults, body weight variability may substantially reflect underlying subclinical illness, frailty, declining functional status, or other health deterioration rather than having a direct causal effect on mortality. Residual confounding due to unintentional weight change could not be fully excluded, and intentional and unintentional weight loss could not be distinguished. Although we excluded individuals who died within one year of the index examination, potential reverse causation may still have influenced the observed associations. Third, body weight or BMI is a limited indicator of adiposity, as it does not account for body composition, fat distribution, or sarcopenia, which can have significant metabolic and prognostic implications for older adults^34^.

Fourth, information on factors that may influence body weight variability—such as changes in diet, or medications affecting body weight—was not available. Lastly, because this study used health screening data from the NHIS, body weight variability was calculated using measurements obtained at one- to two-year intervals. Therefore, short-term fluctuations in body weight may not have been fully captured, potentially leading to an underestimation of the true variability.

Despite these limitations, a major strength of our study is the use of a comprehensive nationwide database that includes health information covering almost the entire population of older adults in South Korea. To our knowledge, this is the first large-scale study to evaluate the association between dynamic body weight variability and both all-cause and cause-specific mortality among community-dwelling older adults, independent of specific disease groups.

## Conclusions

As the aging population continues to grow, the potential clinical relevance of body weight variability in older adults warrants further attention. Taken together, our findings demonstrate that excessive body weight variability is independently associated with an increased risk of all-cause and cause-specific mortality in older adults. This association followed a dose–response pattern and was consistent regardless of the direction of body weight change. These findings suggest that body weight variability may have a potential role as a prognostic indicator of mortality risk in this population.

## Supplementary Information

Supplementary Information 1.

Supplementary Information 2.

Supplementary Information 3.

## Data Availability

The datasets generated and/or analyzed during the current study are not publicly available due to the regulations of the Korean National Health Insurance Service (NHIS) regarding data confidentiality and personal information protection. Access to the database is available only to pre-authorized researchers upon approval by the NHIS.
