# Supplementary material for: Body weight variability and mortality in older adults: a nationwide population-based cohort study
Source: Sci Rep. 2026 May 28;16:24388. doi: 10.1038/s41598-026-55150-3 (PMC13447940; doi:10.1038/s41598-026-55150-3)
Supplement: Supplementary file 1 [file 41598_2026_55150_MOESM3_ESM.docx]

**Supplementary Table S1.** Distribution of VIM values according to quartiles

| Quartile^a^ | VIM range | Median (IQR) |
| --- | --- | --- |
| Q1 | ≤ 1.29 | 0.95 (0.79–1.14) |
| Q2 | ≤ 1.89 | 1.60 (1.43–1.74) |
| Q3 | ≤ 2.76 | 2.26 (2.09–2.50) |
| Q4 | > 2.76 | 3.60 (3.10–4.50) |

VIM, variability independent of the mean; IQR, interquartile range.

^a^Quartiles were defined based on the distribution of VIM values within the study population.
